# Selectivity to Translational Egomotion in Human Brain Motion Areas

**DOI:** 10.1371/journal.pone.0060241

**Published:** 2013-04-05

**Authors:** Sabrina Pitzalis, Stefano Sdoia, Alessandro Bultrini, Giorgia Committeri, Francesco Di Russo, Patrizia Fattori, Claudio Galletti, Gaspare Galati

**Affiliations:** 1 Department of Motor, Human and Health Sciences, University of Rome ‘‘Foro Italico,’’ Rome, Italy; 2 Neuropsychology Center, Santa Lucia Foundation, Rome, Italy; 3 Laboratory of Neuropsychology and Cognitive Neuroscience, Department of Neuroscience and Imaging, University G. d'Annunzio, Chieti, Italy; 4 Institute for Advanced Biomedical Technologies (ITAB), Foundation G. d’Annunzio, Chieti, Italy; 5 Department of Human and General Physiology, University of Bologna, Bologna, Italy; 6 Department of Psychology, Sapienza University, Rome, Italy; Universität Bielefeld, Germany

## Abstract

The optic flow generated when a person moves through the environment can be locally decomposed into several basic components, including radial, circular, translational and spiral motion. Since their analysis plays an important part in the visual perception and control of locomotion and posture it is likely that some brain regions in the primate dorsal visual pathway are specialized to distinguish among them. The aim of this study is to explore the sensitivity to different types of egomotion-compatible visual stimulations in the human motion-sensitive regions of the brain. Event-related fMRI experiments, 3D motion and wide-field stimulation, functional localizers and brain mapping methods were used to study the sensitivity of six distinct motion areas (V6, MT, MST+, V3A, CSv and an Intra-Parietal Sulcus motion [IPSmot] region) to different types of optic flow stimuli. Results show that only areas V6, MST+ and IPSmot are specialized in distinguishing among the various types of flow patterns, with a high response for the translational flow which was maximum in V6 and IPSmot and less marked in MST+. Given that during egomotion the translational optic flow conveys differential information about the near and far external *objects,* areas V6 and IPSmot likely process visual egomotion signals to extract information about the relative distance of objects with respect to the observer. Since area V6 is also involved in distinguishing object-motion from self-motion, it could provide information about location in space of moving and static objects during self-motion, particularly in a dynamically unstable environment.

## Introduction

Analysis of visual motion has a crucial biological significance, in that it allows an animal or a human being to predict the visual trajectory of moving objects so to allow their grasping or avoid potentially dangerous contact with approaching entities. One of the main challenge for the visuo-motor system is to recognize whether a movement signaled at retinal level is due to a true object displacement or is generated by a movement of the subject itself within an otherwise static environment. When moving around the environment we integrate visual, somatosensory, auditory and vestibular cues that allow us to determine and monitor, among other parameters, the speed and direction in which we are heading. Optic flow is probably the most important visual cue for perception of self-motion or ‘egomotion’ (i.e. the sensation to be moving in space) and its neural representation has been extensively studied in humans and macaques.

Several neuroimaging studies have investigated the neural bases of egomotion. Early studies mainly focused on dorsolateral motion areas MT and MST (e.g. [1], [2]). While some studies showed positive evidence in favor of a role of MST in egomotion (e.g., [2]), other failed (e.g., [3], [4]) and the involvement of MST in egomotion perception has been recently questioned [4]. Differential responses to egomotion-compatible optic flow have been recently described in the medial motion area V6 [5], [6], in the cingulate sulcus visual area (CSv: [4]), and in the putative human area VIP [6]. In particular, area V6 was found to be highly selective for optic flow and to respond well to unidirectional motion. In spite of all these studies, the specific role of different cortical regions in recognizing different visual egomotion signals has not yet been determined because their peculiar sensitivity to different types of egomotion-compatible optical flows has never been tested. Egomotion can be experienced along different planes and cardinal axes depending on the *type* of self-movement [2], [7], [8]. Indeed the flow patterns coming to our visual system will be different if we are walking, dancing or moving by car. The optic flow that is generated when a person moves through the environment can be locally decomposed into several basic components, including radial, circular, translational and spiral motion (see [9] for planes and cardinal axes nomenclature). Since coherent circular, translational and radial motion of a wide-field image can specify the cardinal components of observer movement and can produce compelling, illusory self-motion perception (i.e., vection), their analysis must play an important part in the visual control of locomotion and posture. Hence, it is likely that some brain regions in the primate dorsal visual pathway are specialized to distinguish among them. Though no single component is sufficient to represent visual egomotion perception in a comprehensive way, the majority of the neuroimaging studies considered only one type of visual motion component (mainly radial or circular: but see [2], [10], [11].

The aim of this study is to explore the sensitivity to different types of egomotion-compatible visual stimulations in the human motion-sensitive regions. To this aim we used an event-related fMRI experiment, brain mapping methods, and wide-field stimulation. Specifically, we used as visual stimuli ‘*wide-field star fields’* instead of the classic two dimensional patterns of dot, designed to add the depth to the visual stimulation, to give the impression of different types of egomotion in three-dimensional space (such as radial, translational, circular and spiral). Importantly, we performed preliminary psychophysical experiments to verify and quantify vection sensation evoked by the different type of visual stimuli, a check that was often not considered in this type of experiments.

We used dedicated functional localizers both to map the position of area V6 [5] and to distinguish MST+ from MT in each individual subject [12], [13]. The functional responses observed in V6, MT and MST+ have been studied together with those of other motion areas (V3A, CSv and a parietal region likely corresponding to human VIP but hereafter generically called IPSmot because of homology uncertainty) which have been recently found to be involved in the computation of egomotion [4], [6], [14].

Our results revealed that areas V6 and IPSmot mot do discriminate between the various types of coherent motion, areas MT and V3A were not affected by the various types of optic flow, and MST+ showed an intermediate behavior. Area CSv was weakly activated by coherent motion but robustly inhibited by random motion and static stimuli. The role played by V6, IPSmot, and MST+ in computing egomotion is discussed.

## Materials and Methods

### Subjects

A total of 13 subjects (with normal or corrected to normal vision) participated in this study (4 women). Mean age was 28 years (ranging from 23 to 35). All participants gave their informed written consent prior to the scanning session. All procedures were approved by the independent ethic committee of the IRCCS Santa Lucia Foundation of Rome and were performed in accordance with ethical standards laid down in the 1964 Declaration of Helsinki. Each subject participated in 4–5 fMRI imaging sessions. All subjects participated in the main event-related experiment and underwent localizer scans, while 10 subjects participated in the phase-encoded retinotopic experiment. Before scanning, subjects were allowed, if they desired, to consume caffeinated beverages in order to better maintain alertness during the scan session.

### Visual Stimuli and Experimental Design

#### Main fMRI experiment: Three-dimensional flow fields (3D-FF)

**Figure 1:**
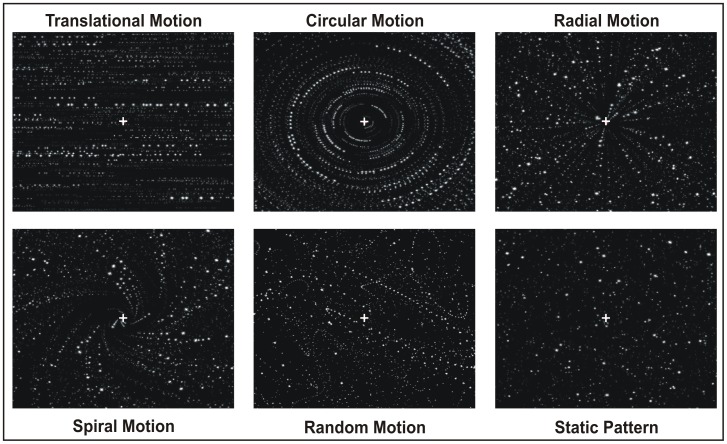
Schematic representation of the stimuli we used in the main fMRI experiment (ff-3D).

In the main event-related fMRI experiment, hereafter called 3D-FF experiment, we separately investigated five different motion components: radial, circular, translational, spiral and random motion. To this aim we created a *wide field* visual display of moving dots with a flow structure that was able to evoke different types of egomotion. Our stimuli were “star fields” composed by high-contrast light dots on a dark background, three-dimensionally designed to give the impression to the observers to be moving in the 3D space. Both speed and size were logarithmically scaled with eccentricity (i.e., as a function of the distance from the center of the display) as when a person moves through a 3D environment. Most previous imaging studies have used displays filling only the central visual field, which do not recreate the wide-field motion typically found in a real scene. The extent of the stimulus field is a crucial parameter which determines the intensity of vection (e.g., [15], [16]). Here, with a wide field stimulation, the subject felt to be immersed in the flow patterns and experienced vivid sensations of egomotion as those experienced in a 3D environment. Star fields simulated various flow patterns, shown in Figure 1 and described as follows:

In the *translational* motion condition, all dots moved horizontally, rightward or leftward, with a different speed according to the different dot size (i.e. the different apparent distance), simulating an observer translating horizontally (such as when a person looks through the window of a moving train).

In the *circular* motion condition, all dots moved in concentric circular paths to produce an impression of clockwise or counterclockwise rotation, consistent with rotation of the observer around the line of sight.

In the *radial* motion condition, all dots moved outwards or inward along the radii of an hypothetical circle to produce an impression of expansion or contraction, respectively. This flow pattern is consistent with movement of the observer forward or backward along the line of sight.

In the *spiral* motion condition, the dots moved as in the radial flow field condition with an added rotational component, resulting in a spiral motion. The rotational component was either clock- or counterclockwise and the global flow pattern changed over time.

Global patterns of optic flow were produced by controlling the local motion directions of the dots. In the four coherent 3D flow patterns, dot patterns inverted direction every 500 ms along either radial (in and out), circular (cw and ccw), translational (left and right) or spiral (cw/in and ccw/out) trajectories. The fast inverting time along a specific direction was chosen to be identical to that used in the flow fields stimulus used to localize area V6 [5].

In the *random* condition, the moving dots changed their local direction and speed at random (every 500 ms), like the random movements of a fly in a 3D box. The purpose was to provide a condition in which local motion was present in all directions and at all locations in a 3D environment, with no global flow structure (incoherent 3D motion).

Original observations in macaque showed the presence in the motion areas V6, MT and MSTd of classes of cells responding to different speeds, from very low (about 1°/s) to very high (more than 100°/s; [17]–[21]). Thus, to optimally activate these motion areas, we used a range of velocities instead of a single speed. In the attempt to activate as many speed-sensitive neurons as possible with a single stimulus we used three different average velocities of about 18°/s, 30°/s and 50°/s.

Since the speed of our stimuli were logarithmically scaled with eccentricity, to simulate real depth, in the radial and spiral motion conditions each individual dot had to have increasing radial speed from the center to the periphery. The following three speed/acceleration conditions were used: dots accelerating from 1 to 20°/sec (average speed 18°/sec), from 5 to 50°/sec (average speed 30°/sec) and from 10 to 70°/sec (average speed 50°/sec). Also, the dot size was directly proportional to the Euclidean distance from the center of the screen, and subtended 0.1 to 4°. In the circular and translational motion conditions, each individual dot was of constant size and speed, but the sizes and speeds differed accordingly to the dot distance from the center of the display.

In all conditions, each dot traveled along an appropriate trajectory for a ‘limited lifetime’ of 350 ms, after which it disappeared to be ‘reborn’ at a new random position. The appearance of new dots was controlled to maintain a constant dot density (0.04 dots/degrees^2^ on average). The dot number, size, lifetime, and speed were the same in all motion conditions.

Control conditions were also tested in the *static* condition, that consisted of a stationary scene equivalent to the last single frame of the last movie (or motion condition) which thus maintained the spatial structure of the optic flow stimulus. This control condition was used to isolate the motion component. There were also *null trials*, i.e., periods in which only a central cross was displayed on the black background. Null trials constituted the low-level baseline for the study.

Overall, we investigated five motion conditions (radial, circular, translational, spiral and random motion) together with two control conditions (static and null trials) for a total of seven experimental conditions. Trials were 3 s periods showing one of the seven conditions while subjects had to fixate a central red cross of 0.4×0.4°. The intertrial interval was set to zero so that the movies were displayed one after the other to avoid that the brisk switch on-off of the movies could bias the neural response in the regions of interest. The experiment was constituted by 15 trials for each of the seven conditions (5 trials for each of the three speeds) for a total of 105 trials. Except for null trials (presented as a sequence of three consecutive null trials every 18 trials), trials were arranged in a pseudo-randomized order which was different for each scan but fixed across subjects. The three speeds were randomly mixed between trials and collapsed together in the analysis.

#### Localizer scans

In a second set of fMRI experiments, we mapped the three motion areas V6, MT and MST+ using two different kinds of localizer visual stimuli. Each functional localizer comprised eight alternations of two conditions presented in blocks of 32 s each (16s ON vs. 16s OFF).

##### V6 Mapping

Two functional scans were acquired in separate sessions to define the medial motion sensitive area V6 as described in Pitzalis et al. [5] and as currently used in our laboratory. Stimuli were 16-s blocks of coherent dot field motion contrasted with 16-s blocks of scrambled motion. A new field of white dots was generated every 500 ms (dot size 0.4×0.4°). Dots immediately began to move along a trajectory so as to generate a coherent movement on a plane. The motion pattern was chosen randomly for that 500 ms period from a continuum ranging from dilation to outward spiral, to rotation, to inward spiral, to contraction. The center of the movement was jittered from flow to flow, and the speed varied within a small range. During the scrambled period, dots and their movement vectors were generated as during the coherent periods, except that each dot trajectory was rotated by a random angle around the pattern center before execution. This scrambled the coherency of movement (at a given point, dots moved in different directions) but preserved the speed gradient (central dots still moved slower than peripheral dots). The average luminance of the stimulus was 31 cd/m2. This stimulus is freely available from the Sereno’s web site (contact sereno@cogsci.ucsd.edu).

##### MT and MST+ Mapping

MST was defined (and distinguished from MT) using the criterion originally introduced by Dukelow et al. [12] and later on used also in other laboratories [4], [11], [13], [22]. Stimuli consisted in 16-s blocks of high contrast (100%) moving dots alternated with 16-s blocks of stationary dots. Dots moved (15°/s) alternately inwards and outwards along the radial axes (thus creating alternating contraction and expansion). Inwards and outwards motion were alternated every 2 seconds. The dots (0.4×0.4°) were restricted to a peripheral circular aperture (15° diameter) presented with its centre placed 10° to the left or right of fixation. Stimuli were restricted to either the left or right hemifield (i.e., one scanning run was completed with the stimulus on the left, and another with it on the right). These peripheral moving stimuli would be expected to evoke neuronal activity in the contralateral hemisphere in both MT and MST, but they would be expected to evoke activity in the ipsilateral hemisphere only in MST, where the receptive fields are large enough to extend into the ipsilateral hemifield (e.g., [12], [13]). Hence, with this procedure, MT and MST can be differentiated in terms of the absence or presence of ipsilateral responses, respectively. However, a limit of the MST definition introduced by Huk et al [13] is that at least four monkey areas (MSTv, MSTd, FST and LST) have the properties used to define MST (ipsilateral representation and motion sensitivity; e.g., [23]). Thus, what is labeled here MST is likely a mosaic of cortical areas and for these reasons hereafter we will call this region MST complex (or MST+).

#### Retinotopic mapping

In a third experiment, we mapped the retinotopic organization of the cortical visual areas in ten out of 13 subjects, using phase-encoded stimuli, as described elsewhere [5], [23], [24], [25], [26]. Shortly, the stimuli consisted of high-contrast flickering colored checks in either a ray- or a ring-shaped configuration (polar angle and eccentricity, respectively). These stimuli spared a central 0.75° circular zone of the visual field to avoid ambiguities caused by fixation instability. Periodic stimuli moved slowly and continuously, and checks reversed between bright and dark at a rate of 8 Hz (64 s/cycle, 8 cycles/scan). The average luminance of the stimuli was 105 cd/m2.

### Apparatus and Procedures

The MR examinations were conducted at the Santa Lucia Foundation (Rome, Italy) on a 3T Siemens Allegra MR system (Siemens Medical Systems, Erlangen, Germany) equipped for echo-planar imaging. Single-shot echo-planar imaging (EPI) images were collected using blood-oxygenation-level-dependent imaging [27] by a standard transmit-receive birdcage head coil.

Stimuli were generated by control computers (a standard PC and an SGI O2, both equipped with a standard 3D graphics card) located outside the MR room and running different software for each specific experiment. For the FF-3D experiment, stimuli were presented with an in-house software, implemented in MATLAB (The MathWorks Inc., Natick, MA, USA) using Cogent 2000 (developed at FIL and ICN, UCL, London, UK) and Cogent Graphics (developed by John Romaya at the LON, Wellcome Department of Imaging Neuroscience, UCL, London, UK). For the V6 localizer scan and the retinotopic mapping, stimuli were presented with an in-house OpenGL program (Mapper software) code by A. Dale and M. Sereno. For the MST localizer scan, stimuli were presented with Presentation 9.9 (Neurobehavioral System In., Albany, Canada) code by A. Bultrini.

In all experiments we used a wide-field stimulation (up to 82° in total visual extent). To get a wide field stimulation in the scanner bore, we substantially changed the standard set-up [26]. Visual stimuli were projected by an LCD video projector with a customized lens to a back projection screen attached on the posterior border of the head coil, reducing the average viewing distance to 20 cm. We had to lower down the subject's body of about 4 cm to not cover the bottom portion of the screen and use an enlarged mirror so that also the most peripheral part of the screen could be viewed. This setup allowed a large field of view also in the 3T magnet. At this short distance, visual stimuli subtended up to 69° (±34.5) horizontally, 55° (±27.5) vertically, and 82° (±41) in an oblique direction in the visual field. In addition to better reveal areas that emphasize the periphery, the wide field stimulation is particularly indicated in those studies (as the present one) where one wants to evoke in the observer a vection sensation, that is the illusion of egomotion induced by optic flows. Using a wide field stimulation the subject felt to be immersed in the flow patterns and induced vection was particularly compelling as revealed by the psychophysical experiment (see behavioral results). Fixation distance and head alignment were held constant by a chin rest mounted inside the head coil. Subjects’ heads were stabilized with foam padding in order to minimize movement during the scans. All experiments used passive viewing and subjects were required to gaze at a central cross throughout the period of scan acquisition. The wide-field visual projection setup did not allow for eye-tracking. However, to promote stable fixation during all conditions the fixation point was continuously visible at a fixed position on the screen and only expert subjects with a good fixation stability were used.

#### Imaging parameters

For the 3D-FF experiment and localizer scans, MR 30 axial slices were 4 mm thick (with a 0 mm gap, interleaved excitation order), with an in-plane resolution of 3×3 mm, oriented approximately to the AC-PC line. From the superior convexity, sampling included almost all the cerebral cortex, excluding only the ventral portion of the cerebellum.

Each participant underwent six consecutive scans for the 3D-FF experiment and four scans for the localizer. Each scan took either 324 s (3D-FF) or 256 s (localizer scans) with 162 or 128 single-shot EPI images per slice, respectively. Retinotopic mapping was acquired in a separate day using the same apparatus, setup, and coil as for the main experiment. Images were acquired as in the main experiment, but in this case MR slices (4 mm thick, with an in-plane resolution of 3×3 mm) were oriented approximately parallel to the calcarine fissure and covering only the posterior part of the brain. Part of the retinotopic data were acquired with slices having a smaller thickness of 2.5 mm and oriented approximately parallel to the calcarine fissure (thus covering all the brain). This voxel size strikes a compromise between sufficient signal-to-noise and the ability to assign activations to the proper sides of the sulci and gyri. To increase signal to noise, data were averaged over three runs for each stimulus type (eccentricity and polar angle). Thus, in total, each participant underwent six scans for the retinotopic mapping. Each scan took 512 s, with 256 single-shot EPI images per 32 contiguous slices. Other standard imaging parameters were in common between experiments (TR = 2 s, TE  = 30 ms, TA  = 66.6 ms, flip angle  = 70°, 64×64 matrix, bandwidth = 2298 Hz/pixel). Overall, a total of 190 scans were carried out on the 13 subjects (78 scans for the FF3d experiment, 52 scans for the functional localizer and 60 scans to map retinotopic visual areas).

In each scan, the first 8 seconds of the acquisition were discarded from data analysis in order to achieve a steady state, and the experimental tasks started at the beginning of the fifth volume. The cortical surface of each subject was reconstructed from two to three structural scans (T1-weighted Magnetization Prepared Rapid Gradient Echo, MPRAGE, sequence, TR  = 2.00 s, TE  = 4.38 ms, flip angle  = 8°, 1×1 mm in-plane resolution, matrix 256×256, 176 contiguous 1 mm thick sagittal slices, bandwidth  = 130 Hz/pixel). The last scan of each functional session was an alignment scan (also MPRAGE, 1x1x1 mm) acquired in the plane of functional scans. The alignment scan was used to establish an initial registration of the functional data with the surface. Additional affine transformations that included a small amount of shear were then applied to the functional scans for each subject using blink comparison with the structural images to achieve an exact overlay of the functional data onto each cortical surface.

### Psychophysical Validation

Earlier studies on the neural bases of egomotion have not tested whether the visual stimulation actually induced self-motion. They used an *egomotion-consistent* stimulation [6], [22], or assumed that the applied coherent moving stimulus resulted in vection (e.g., [16], [28]. However, because both the duration and the subjectively estimated strength of vection show large interindividual differences [29], it is difficult to relate their results to the self-motion sensation per se. Therefore, in this study, we have preliminarily performed a psychophysical experiment to verify and quantify vection sensation evoked by the different type of visual stimuli we were going to use into the bore.

A group of 15 subjects (5 males; mean age was 22 years, ranging from 20 to 26) underwent a psychophysical session aimed at quantifying the perceived self motion sensation evoked by the experimental stimuli used in the main experiment. Visual stimuli were presented on a 17′ computer display that subtended the same degrees of visual angle as in the fMRI scanner. Subjects were seated in front of the display in complete darkness, with the head mechanically stabilized with a chin rest and a head holder. Subjects viewed the same stimuli as in the main experiment in a randomized sequence, and answered to the following question immediately after viewing each movie: “How intense was the sensation that you were moving in the space?” (self motion sensation: SMs). Participants indicated the intensity of SMS through a Visual-Analog Scale (VAS). The VAS was shown on the screen as a 10-cm white horizontal line on a dark background intersected by a small vertical mark. Subjects made the mark slide along the horizontal line by using the computer mouse, and clicked when the mark was located at the point they felt to correspond with the subjective intensity of their sensations. The left and right ends of the horizontal line represented no sensation at all and maximal sensation, respectively. The VAS score was determined as the distance (in cm) of the mark from the left end of the line, and thus ranged from 0 to 10.

### Data Analysis

#### Anatomical image processing

FreeSurfer was used for surface reconstruction [30], [31]. High resolution structural images obtained from each subject were manually registered and averaged. After reconstructing each hemisphere, we completely flattened the inflated occipital lobe after first cutting it off posterior to the Sylvian fissure, and making an additional cut along the Calcarine fissure. Stereotaxic coordinates were calculated through an automatic nonlinear stereotaxic normalization procedure [32], performed using the SPM8 software platform (Wellcome Department of Cognitive Neurology, London, UK), implemented in MATLAB (The MathWorks Inc., Natick, MA, USA).

#### Functional image processing: main experiment and localizer scans

Images from the main experiment and functional localizers were preprocessed and analyzed using SPM8 (Wellcome Department of Cognitive Neurology, London, UK). Functional time series from each subject were first temporally corrected for slice timing, using the middle slice acquired in time as a reference, and then spatially corrected for head movement, using a least-squares approach and six-parameter rigid body spatial transformations. They were then spatially normalized using an automatic nonlinear stereotaxic normalization procedure (final voxel size: 3×3×3 mm) and spatially smoothed with a three-dimensional Gaussian filter (6 mm full-width-half-maximum). The template image for spatial normalization was based on average data provided by the Montreal Neurological Institute [33] and conforms to a standard coordinate referencing system [34]. The time series of functional MR images was first analyzed separately for each participant. The effects of the experimental paradigm were estimated on a voxel-by-voxel basis, according to the general linear model. The onset of each trial constituted a neural event, which was modeled through a canonical hemodynamic response function, chosen to represent the relationship between neuronal activation and blood flow changes. Separate regressors were included for each trial type (radial, translational, circular, spiral, random, static and fixation), yielding parameter estimates for the average hemodynamic response evoked by each trial type. The model also included a temporal high-pass filter, to remove low-frequency confounds with a period above 128 s. Serial correlations in the fMRI time series were estimated with a restricted maximum likelihood (ReML) algorithm using an autoregressive AR(1) model during parameter estimation, assuming the same correlation structure for each voxel, within each run. The ReML estimates were then used to whiten the data. These subject-specific models were used to compute a set of contrast images per subject, each representing the estimated amplitude of the hemodynamic response in one trial type relative to the fixation baseline. Contrast images from all subjects were entered into a within-subjects ANOVA with non-sphericity correction, where subject was considered as a random effect, thus allowing to draw inferences related to the whole population our participants were extracted from.

We used the model described above to search the whole brain for regions differentiating any of the five motion types (translational, circular, radial, spiral and random) from the static condition (static frames). The resulting statistical parametric map of the F statistics was thresholded at the voxel level and by cluster size. Correction for multiple comparisons was performed using false discovery rate (p<0.05; extent threshold  = 10 voxels). The resulting regions include all voxels showing a reliable BOLD response during motion vs. static frames, irrespective of the kind and amount of motion and of the sign (positive or negative) of the evoked BOLD response. In-house software (BrainShow, written in Matlab) was used to visualize the resulting regions onto a population-average, landmark- and surface-based (PALS) atlas [35], and to assign anatomical labels to activated areas at the level of Brodmann areas and cortical gyri. Brodmann areas were derived from the Talairach Daemon public database [36], while cortical gyri were derived from a macroscopical anatomical parcellation of the MNI single-subject brain [37]. BrainShow has been used in previous studies from our and other groups (e.g. [5], [38], [39], [40], [41]) and is freely available on request for academic usage (E-mail: gaspare.galati@uniroma1.it ).

After identifying the regions differentiating motion from static frames, we searched for modulation of BOLD responses in these regions as a function of the motion type. This step was performed on regionally averaged data as follows. Regions were defined as clusters of significantly activated adjacent voxels at most 8 mm away from each local maximum of the group statistical map. For each subject and region, we computed an estimate of the averaged amplitude of the hemodynamic response in each experimental condition, by entering a spatial average (across all voxels in the region) of the pre-processed time series into the individual general linear models. Such regional hemodynamic response estimates were then analyzed through a repeated-measures analysis of variance (p<0.05). The *post-hoc* Duncan’s test was performed when appropriate (p<0.05). Note that, although the analysis used to define the regions and the selective analysis conducted on the regionally averaged data are based on the same dataset, they are inherently independent. The first step tests for the presence of any motion-related neural response regardless of the kind of moving stimulus, while the second step tests for modulations induced by the kind of moving stimulus, thus avoiding the risk of “double dipping” [42].

We also performed the same regional analysis on the visual regions V6, MT and MST+, as individually defined on the basis of the independent localizer scans as described in previous studies. For localizer scans, stimulation blocks were modelled as box-car functions spanning the whole block duration and convolved with a standard hemodynamic response function. We used individually adjusted uncorrected thresholds to define these regions (p<0.001 or higher). To define the V6 ROI we used the same procedure described in our previous paper [5]. To define the MT/MST+ ROI we followed the same procedure described several times in previous papers (e.g., [4], [11], [12], [13], [22]) that can be summarized as follows. As contralateral stimuli drive the entire MT+, and ipsilateral stimuli drive only MST+, the activation regions obtained for the left and right stimuli overlapped substantially. MST+ was defined as all contiguous voxels that were significantly active during ipsilateral motion stimulation. MT was defined as all contiguous active voxels that were active during contralateral but not ipsilateral stimulation, with one proviso. As previous research [11], [12], [13] has shown that the centre of MST+ is located anteriorly with respect to the centre of MT, any MT voxels situated further anterior than the median value of MST+ ROI on the horizontal (axial) plane were removed from the MT ROI as done also in other previous studies (e.g., [22]).

#### Motion Coherence Coefficient

To quantify the differential sensitivity of each region to the motion coherence as evoked by our visual stimulation, we extracted from each ROI (by averaging across all voxels in a ROI) the effect sizes for coherent and incoherent (i.e. random) motion conditions. We averaged together the results across the four coherent conditions to get a single value to be assigned to the coherent motion. We then computed the ratio of the means MC (Motion Coherence) and MI (Motion Incoherence) for each ROI and averaged the results across hemispheres. A MC/MI coefficient of 1 means that MC and MI have the same effect in a given ROI, a value smaller than 1 means that MI has a greater effect than MC whereas a value bigger than 1 means that MC has a greater effect than MI (for a similar approach see [6]).

#### Analysis of retinotopic data

Retinotopic data were analyzed using FreeSurfer [30], [31] based on standard procedures described in details in many previous studies (e.g., [5], [24], [25], [26], [43]). Briefly, P values were estimated on a voxel-by-voxel basis by constructing an F ratio between ‘‘signal’’ (response amplitude at stimulus frequency) and ‘‘noise’’ (amplitude at other frequencies excluding second and third harmonics) with degrees of freedom equal to the number of time points. The phase of the signal at the stimulus frequency was used to map retinotopic coordinates (polar angle or eccentricity). The boundaries of the retinotopic visual areas were defined in each participant on the basis of the field-signs calculated from the maps of polar angle and eccentricity [24].

## Results

### Behavioral Results

**Figure 2:**
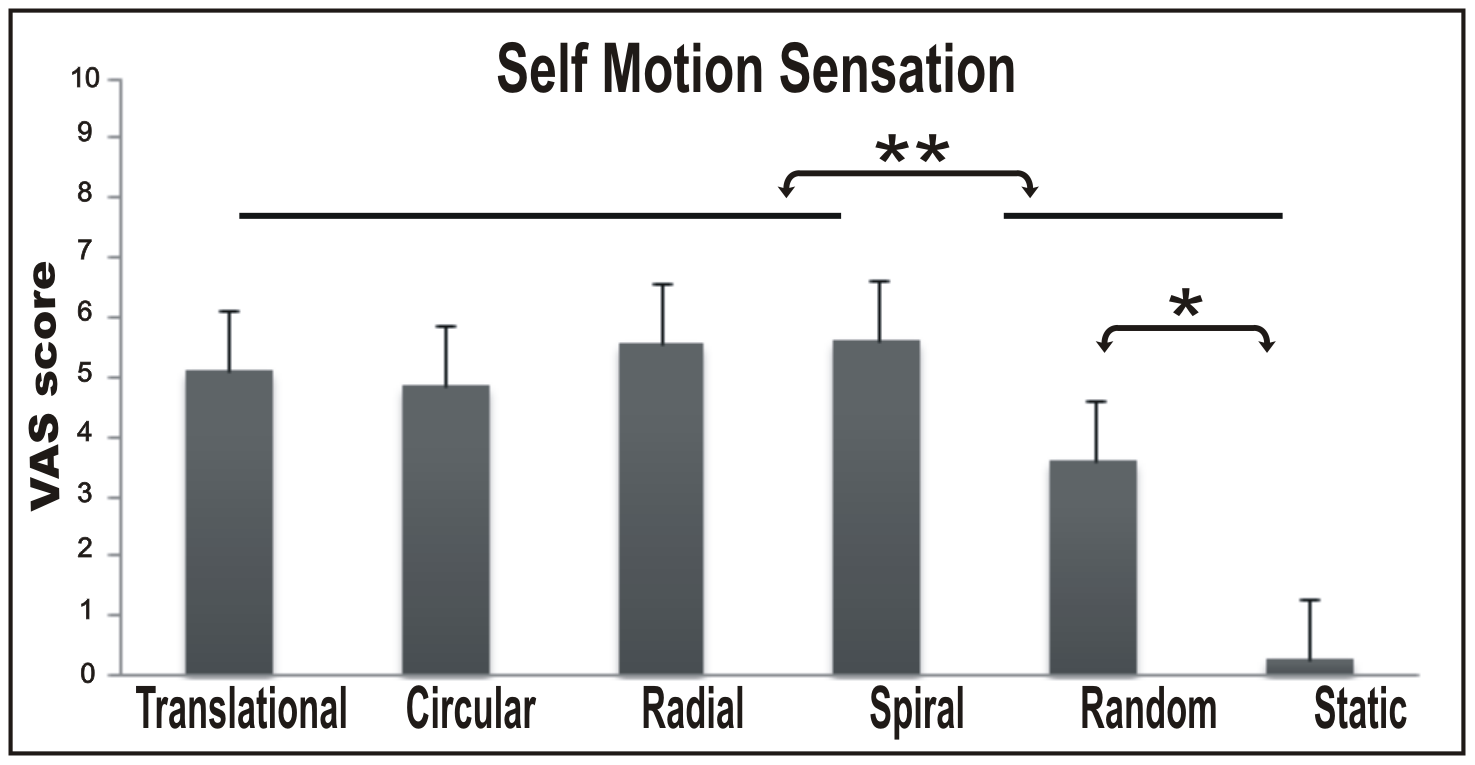
Psycophysical results. Histograms show the Intensity of self motion sensation revealed by VAS scale across subjects. Bars represent the mean VAS scores ± standard error of the mean across runs and participants.

Figure 2 shows rates, averaged across subjects, for self-motion sensation (SMs) in the six experimental conditions (translational, circular, radial, spiral, random, and static) as assessed during the preliminary behavioral validation. The data were submitted to a one-way repeated measures ANOVA with experimental condition as factor. The analysis was significant (SMs: F_(5,70)_  = 20.16, p<0.001).

Post-hoc comparisons revealed that subjects perceived a stronger self motion experience for the four coherent 3D motion stimuli (with no differences among them) than for random motion (p<0.05) and static stimuli (p<0.0001). Moreover, the SMs in the random condition was higher than in the static condition (p<0.001), which was near zero. Overall, the four coherent motion conditions were all able to evoke in the subject a strong self-motion sensation but each along a different plane.

### Imaging Results

**Figure 3:**
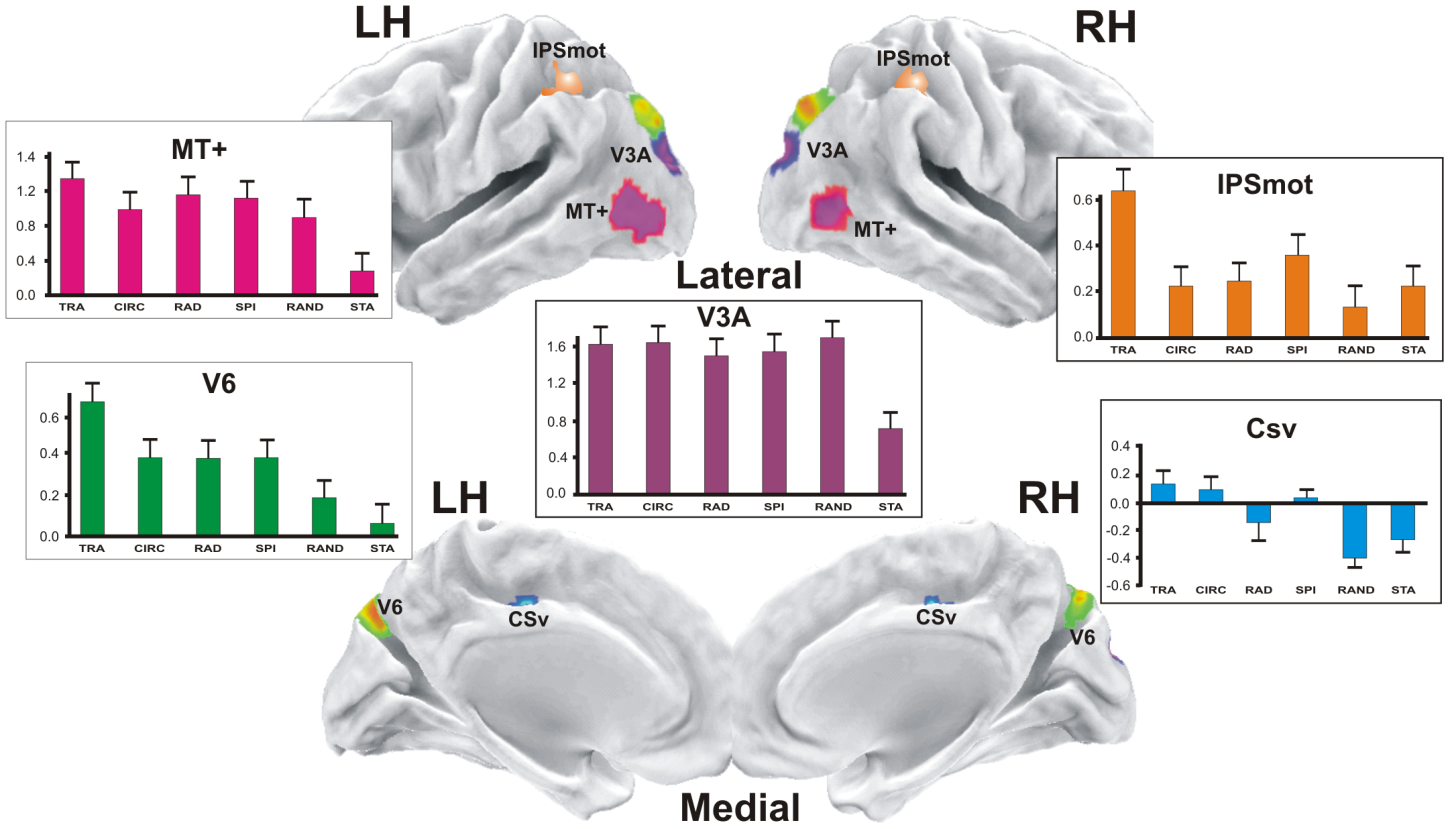
Motion areas. In color are the regions more activated in at least one of the motion (M) conditions relative to the static (S) condition (contrast M-S): V6; *MT+*, middle temporal complex; V3A; *CSv*, visual cingulate sulcus area; IPSmot, Intra Parietal Sulcus motion area. The plots represent the averaged BOLD percent signal change ± standard error of the mean across subjects and hemispheres for each experimental condition labelled as follows: TRA, translational; CIR, circular; RAD, radial; SPI, spiral; RAND, random; STA, static.

In order to reveal general differences in cortical areas specifically associated to the motion conditions, as a first step we selected regions showing greater fMRI responses in at least one of the motion (M) conditions relative to the static (S) condition (contrast M-S). The rationale behind this approach is that we first wanted to isolate the areas responding to motion from other visual areas responding to the physical presence of the stimulus per se (i.e., sensorial response in early visual areas). Results from the contrast M-S, shown in Figure 3, revealed significant bilateral activations in five cortical regions (putatively, areas V6, MT+, V3A, CSv and IPSmot) which are displayed together on the semi-inflated cortical surface reconstruction of the left and right hemispheres of the average brain. The parietal region (orange ROI) could correspond to the human area VIP. However, four different locations have been obtained for VIP in humans by Bremmer et al. [44], Cardin and Smith [6], Bartels et al. [45] and Sereno and Huang [46]. Thus, given that in absence of monkey fMRI data the homology question cannot be settled, we choose the neutral name of Intra-Parietal Sulcus motion region (IPSmot).

**Figure 4:**
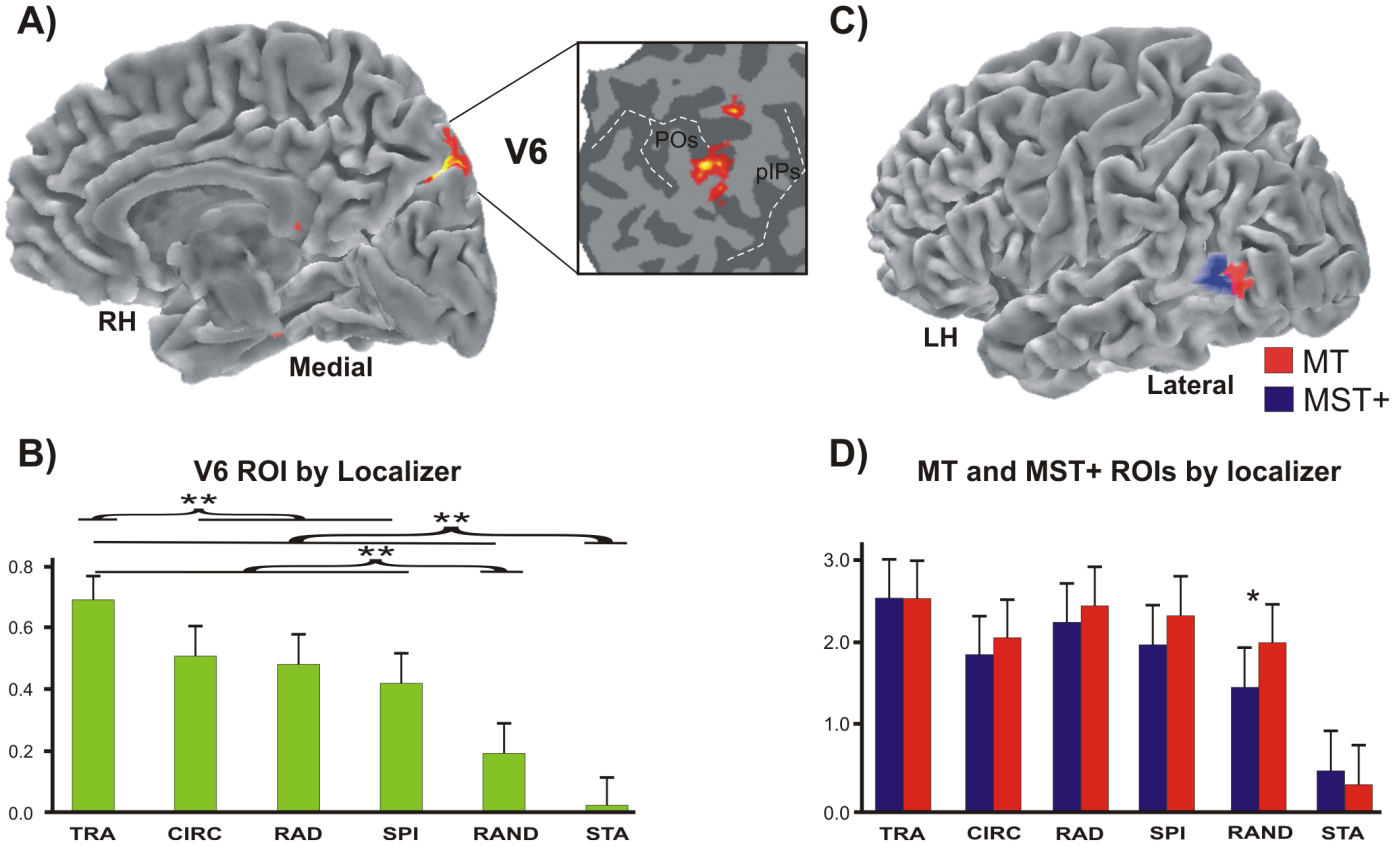
Motion areas mapped by localizers. (A) Area V6 mapped with the functional localizer, i.e., coherent flow versus randomly moving dots. Results are displayed on the medial folded representation of the right hemisphere of the template brain. The anatomical location of V6 can be better appreciated in the close-up of the flattened surface (white box) taken from the same average brain. Dashed lines, fundus of the main sulci; POs, parieto-occipital sulcus; pIPs, posterior end of the intraparietal sulcus. (B) Plots represent the averaged BOLD percent signal changes ± standard error of the mean in the localizer-defined area V6. (C) Imaging results from the functional localizer used to map areas MT and MST+ (i.e., ipsilateral vs contralateral radial motion). Results are displayed on the lateral folded representation of the left hemisphere of the template brain. (D) Plots represent the averaged BOLD percent signal changes ± standard error of the mean in the localizer-defined areas MT and MST+.

As a second step, we determined the objective position of areas V6, MT, and MST+ in each individual subject using the dedicated functional localizers as described in the Methods (see Figures 4A and C, respectively).

**Figure 5:**
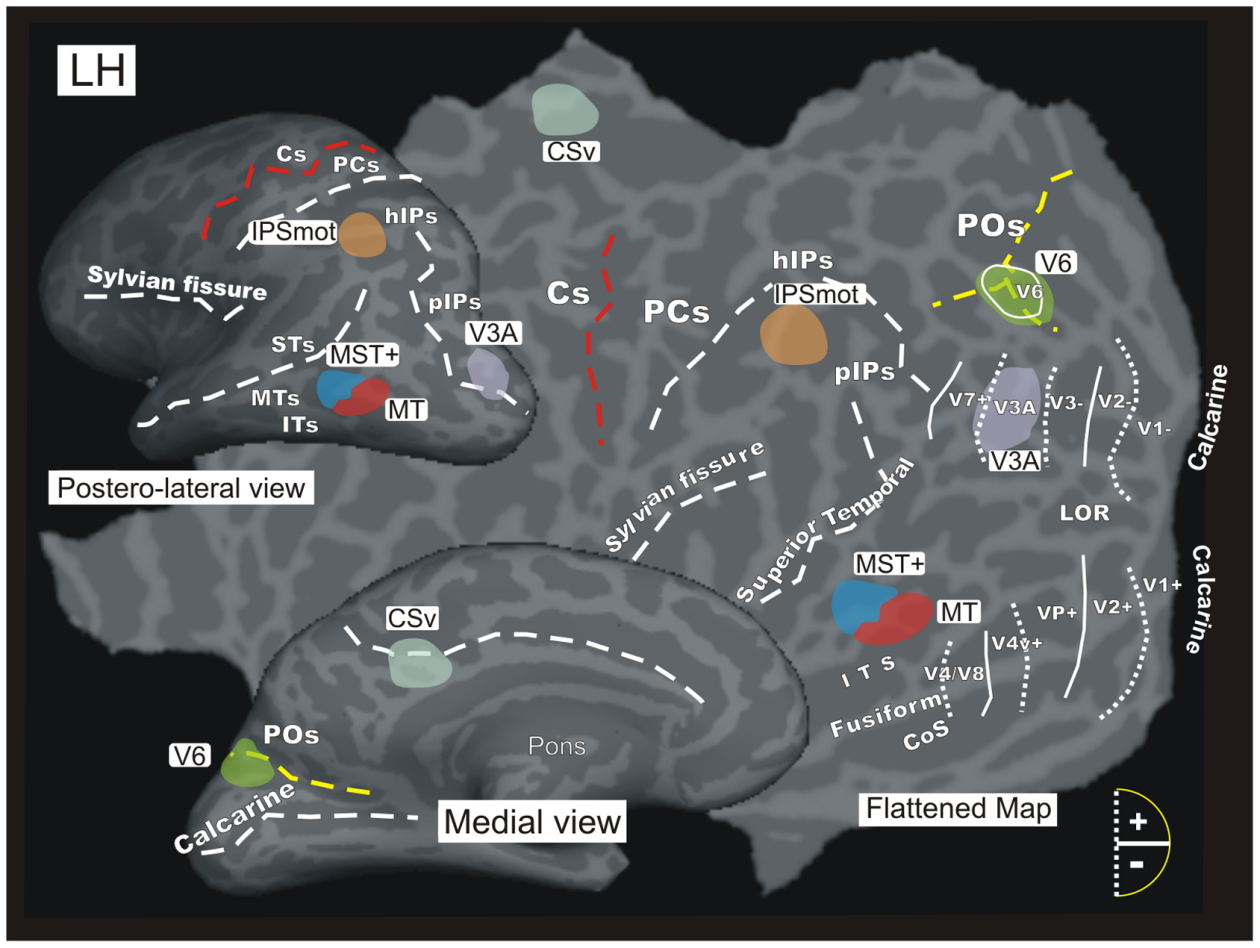
Motion and other visual areas of the brain. An inflated (medial and dorso-lateral views) and flattened representation of the left hemisphere of a representative participant marked with the locations of the six average regions of interest (ROIs) that were studied: V6, MT, MST+, V3A, CSv and IPSmot. The six ROIs are displayed together with the borders of the visual areas identified in this subject by retinotopic mapping. Area V6 as defined by the retinotopic mapping is indicated by white outline and label on the POs, which overlaps with the V6 ROI in green. The dashed lines reported on both the flat map and the inflated representations indicate the fundus of the major sulci. Dotted and continuos lines indicate the vertical and horizontal meridian representations in visual areas, respectively. Major sulci (dark gray) are labeled as follows: ITs, Inferior Temporal sulcus; MTs, Middle Temporal sulcus; STs, Superior Temporal sulcus; hIPs, horizontal segment of the intraparietal sulcus; PCs, post-central sulcus; Cs, Central sulcus; LOR, Lateral Occipital Region; COs, Collateral sulcus. Other labels are as in Figure 4.

As a third step, a retinotopic mapping was performed to illustrate the exact relationship between the activated motion regions and the known early visual cortical areas. In Figure 5 all the functionally identified average regions are displayed in color on the left hemisphere of a representative participant, together with a set of lines representing the boundaries of early visual areas in this same subject obtained with the retinotopic mapping analysis. This overlay enabled us not only to confirm the ‘identity’ of some specific functional areas (as V6 and V3A), but also to localize the activated regions with respect to the brain cortical sulcal anatomy. This aspect is especially important to compare present results with previous human fMRI studies that used individual approach and anatomical (not only functional) description of the activated regions (e.g., [5], [6], [11], [26], [40]).

As a fourth step, we studied the functional response profile of the resulting motion regions (V6, MT, MST+, V3A, CSv, and IPSmot) in order to explore their sensitivity to different type of egomotion flow patterns. The mean percentage signal changes we observed in the motion conditions relative to the baseline (i.e., fixation) are plotted in the column histograms of Figs. 3, 4B and 4D.

The anatomical and functional definitions of each motion region are described in details below.

#### Area V6

Results showed a region selectively activated by motion stimuli in the dorsalmost part of the parieto-occipital sulcus POs (Fig. 3). The location of this region well corresponds to the location of human area V6 recognized on the basis of a wide-field retinotopic analysis (Fig. 5) [26]. The area found here is indeed located on the dorsal margin of the POs in correspondence of its posterior bank, and has MNI coordinates (x = ±13, y = –81, and z = +44) well compatible with those of the retinotopic V6 [26].

To check whether this region actually corresponds to the medial motion area V6, we independently defined area V6 according to the functional localizer (see methods) in all scanned subjects, i.e., 26/26 hemispheres (Fig. 4A). The map found with the localizer (Fig. 4A) has the following MNI coordinates: x = ±9, y = –82, and z = +36 and its position closely resemble that obtained with the contrast M-S (Fig. 3). As described in our previous paper [5], when using this functional localizer the map of area V6 nicely overlaps with that obtained with wide retinotopic stimulation. This can be clearly appreciated in Figure 5, where area V6 (green) as defined by functional localizer shows a good overlap with the area V6 (white outline) as retinotopically defined in this subject.

The results show that area V6 (as defined by the general contrast M-S) responds more to any type of motion than to static stimuli (p<0.05), and more to coherent than to incoherent (random) stimuli (p<0.05, see green columns plot in Fig. 3). Among the various types of coherent motion, area V6 responds preferentially to the translational motion (p<0.05). V6 is not differentially modulated by circular, radial and spiral motion. Plots in Figure 4B show that area V6 defined by the localizer has a functional profile very similar to that observed in Figure 3 (i.e., it has a great sensitivity to translational motion, p<0.05, while it responds quite strongly but indifferently to the other types of coherent motion). One-way repeated measures ANOVA (F_(5,70)_  = 2.708 p<0.05), and post hoc analysis (p<0.05) gave the same results in statistical terms in the two sets of data. This is particularly important for the reliability of the functional profile of area V6, as we used two independent set of data to draw the V6 ROI. In the V6 ROI as defined by the functional localizer (i.e., coherent vs incoherent motion) the voxels taken into account were belonging to V6 by definition, and data could be biased due to the fact that only those voxels with a preference for the coherent motion were included in the ROI. On the contrary, V6 ROI as defined by the contrast M-S does not suffer from this confound and can be considered ‘unbiased’. The strong preference for the translational coherent motion observed in both the biased and unbiased V6 ROIs highly increases the reliability of the results and again suggest that the V6 as defined by the M-S contrast is the same area as that defined by the localizer.

#### Areas MT+ and its functional subdivisions MT and MST+

Results from the contrast M-S revealed significant bilateral activations in the temporal cortex (Fig. 3). The mean MNI coordinates of this region (x = ±45, y = –71, z = +4; Table 1) are in good agreement with those of the classic motion sensitive region MT+ described in earlier studies using both PET (x = ±42, y = –69, and z = 0; [47]) and fMRI (x = ±45, y =  −76, and z = +3; [1]).

**Table 1:** Mean MNI coordinates of ROIs identified in the present study.

ROIs	MNI coordinates		
	X	Y	Z
V6	±13	−81	44
V6 (localizer)	±9	−82	36
MT+	±45	−71	4
MT (localizer)	±45	−80	−2
MST+ (localizer)	±47	−69	8
V3A	±20	−87	29
CSv	±15	−33	39
IPSmot	±30	−60	45

In the case of V6, MT+, CSv, V3A and IPSmot regions, ROIs were extracted from the statistical contrast (M-S). In the case of V6 (localizer), MT (localizer), and MST+ (localizer), ROIs were extracted from their functional localizers. The table shows the coordinates of the maxima of the motion activated regions (values are in mm). All maxima were significant at p<0.05 (whole brain, FDR corrected).

It is now generally acknowledged that the large motion-sensitive region MT+ is a complex of several areas (e.g., [5], [23]), including areas as MT and MST, which have different functional profiles and could be differently involved in egomotion perception. Previous authors have shown that unlike MT, MST shows a degree of preference for at least a single flow stimulus (e.g., [4]). To check the preference of these two areas to different types of flow stimuli, we mapped MT and MST+ ROIs using an independent functional localizer following standard procedures as described in the Methods and in many previous papers (e.g., [4], [11], [12], [13]). MT was successfully defined in 22/26 hemispheres. Although ipsilateral responses were relatively weak compared with contralateral responses, a subregion of ipsilateral activity was clearly identifiable in 20/26 hemispheres and was marked as MST+. The two regions resulting from this analysis are rendered in Figure 4C. The mean MNI coordinates of MT region are x = ±45, y = –80, and z = −2, those of MST+ region are x = ±47 y = −69 z = 8 (see Table 1). Note that the mean MNI coordinates of MT region closely correspond to those provided in Kolster et al. [23] for the neighboring area pV4t (RH: x = +47, y = –81, and z = −2). Thus, what is labeled MT here could be a mixture of MT and neighboring pV4t according to Kolster et al [23]).

Figure 5 shows that the MT and MST+ ROIs occupy an anatomical position in between the Inferior Temporal sulcus (ITs) and the Middle temporal Sulcus (MTs), which is in line with the description of these two regions provided in previous fMRI studies where the two motion areas have been distinguished [11], [12], [13]. Specifically, area MT (red) was anterior to and distinct from the retinotopically defined areas V1, V2, VP, V4v, V4/V8 whose locations, indicated in the flat map, were previously identified in this subject. Area MST+ (dark blue) was always anterior and often dorsal to MT, although there was some degree of variability across subjects. A constant evidence was that MST+ typically abutted MT, as previously reported by Huk et al. [13].

Purple plots in Figure 3 show that region MT+ (as defined by the general contrast M-S) responds more to any type of motion than to static stimuli (p<0.001) and a bit more to coherent than to incoherent (random) stimuli (p<0.05). Like V6, among the various type of coherent motion MT+ responds preferentially to the translational motion (p<0.05) and is not differentially modulated by circular, radial, and spiral motion. Contrary to V6, however, the response to random motion is high in MT+, being only slightly weaker than that observed for coherent motion (n.s.). This explains why MT+, in contrast to V6, results insensitive to optical flow stimulation when coherent minus incoherent stimulation was used as a paradigm [5].

Red plots in Figure 4D show that the localizer-defined area MT responds more to any kind of motion than to static stimuli (p<0.0001). Area MT distinguishes between random and at least three types of coherent motion, translational (p<0.001), radial (p<0.001), and spiral (p<0.05) with no preference among the three types of coherent motion. Also the fourth type of coherent motion we tested (circular motion) is more effective than random in activating MT, but the difference does not reach statistical significance.

Blu plots in Figure 4D show that the localizer-defined area MST+ responds more to any kind of motion than to static stimuli (p<0.0001), and more to coherent than to incoherent (random) stimuli (p<0.005). MST+ responds more to the translational (p<0.001)and radial motion (p<0.5), which do not differ each other, with the circular motion being the less favorite condition.

To test for functional differences between MT and MST+ ROIs, the two regions were submitted to a 2 by 6 repeated-measures ANOVA with factors Region (MT and MST+) and stimulus type (radial, translational, circular, spiral, random, static), where the five motion conditions and the control static condition were considered as six levels of a single variable. This analysis yielded a significant Region by stimulus type interaction (F_5,95_ = 5.81; p<0.0001), indicating a different sensitivity of MT and MST+ for the different type of stimuli. Post-hoc comparisons revealed that MT responds more strongly to random motion than MST+ (p<0.05), while the two areas did not differ each other respect to the other components.

#### Area V3A

The contrast M-S showed significant activity in the posterior segment of the IPs (Fig. 3). The mean coordinates of this region are x = ±20 y = −87 z = +29 (Table 1). This location corresponds to the position of the retinotopic dorsal visual area V3A, especially in the X and Y directions (e.g., [25]). It does not extend either medially or antero-laterally toward the typical positions of retinotopic dorsal areas V3 and V7, respectively. The activation is located posteriorly and laterally to that of the V6 ROI, bordering its posterior part. Retinotopic mapping confirms that this region is indeed V3A (Fig. 5). In fact, in Figure 5 the motion area V3A (violet) is located on the posterior segment of the IPs (pIPs), a location remarkably coincident with the position of retinotopic area V3A, whose anatomical landmark is indeed the pIPs [25], [48].

Violet plots in Figure 3 show that area V3A responds more to any kind of motion than to static stimuli (p<0.001), but it is not particularly sensitive to the coherence of motion. It has a great response to random motion (which is not statistically different with respect to the coherent motion) and responds equally well to different coherent movements. In other words, V3A responds to motion independently to the presence/absence of coherence in it.

#### Area CSv

The M-S contrast showed significant bilateral activity in the depth of the medial posterior cingulate sulcus (Fig. 3). Figure 5 shows more specifically that the CSv area (light blu) is located in between the posterior ascending portion of the cingulate sulcus (also called the marginal ramus of the cingulate sulcus) and the corpus callosum. This location, as well as the mean coordinates of this region (x = ±15 y = −33 z = +39; Table 1), corresponds well to the original definition of human CSv provided by Wall and Smith [4].

Cyan plots in Figure 3 show that area CSv is weakly, if any, activated by coherent motion (p<0.05), while its activity is consistently suppressed below the baseline by random motion (p<0.005) and static stimulations (p<0.05). This means that CSv does not discriminate between the various types of coherent motion, which indeed evoked BOLD signals that do not statistically differ each other.

#### Area IPSmot

The contrast M-S showed significant activity along the horizontal segment of the IPs (Fig. 3). The mean MNI coordinates of this region (x = ±30 y = −60 z = 45; Table 1) are in line with those of area VIP as described in previous fMRI studies [4], [6].

Orange plots in Figure 3 show that area IPSmot responds more to translational motion (p<0.005) than to every other kind of visual stimulations we used. The area shows also a weaker but significant response to spiral motion with respect to the random condition (p<0.05), followed by circular, radial random and static conditions, which do not statistically differ each other.

### Sensitivity to Motion Coherence

**Figure 6:**
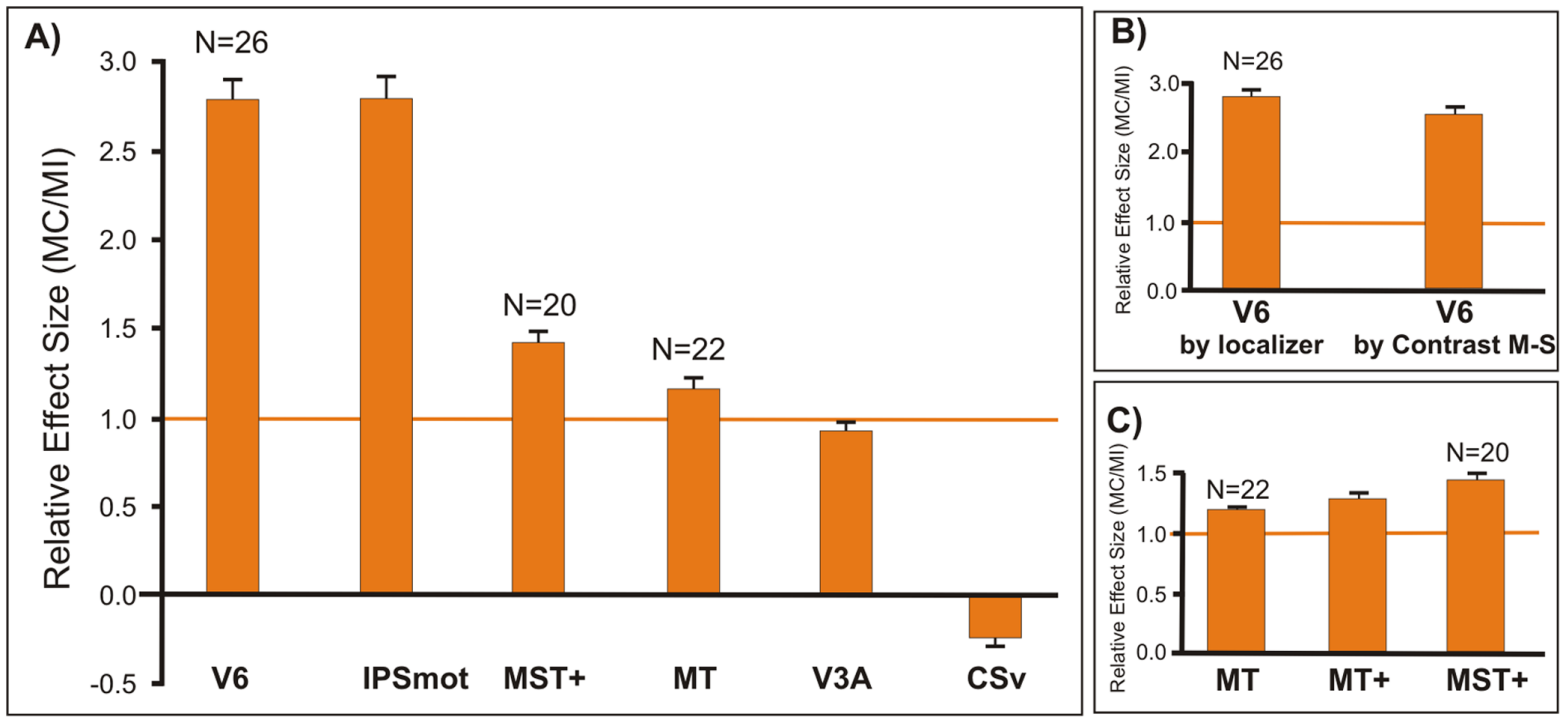
Cortical responses to motion coherency. Average motion coherence coefficients extracted from the functionally defined ROIs (see Materials and Methods). (A) MC/MI coefficient in areas CSv, V3A, and IPSmot, as defined by the group statistical contrast (M-S) and in areas V6, MT and MST+, as defined by functional localizer. (B) MC/MI coefficient in area V6, as defined by the group statistical contrast (M-S) and by functional localizer. (C) MC/MI coefficient in area MT+, as defined by the statistical contrast (M-S) and in its functional subdivisions MT and MST+, as defined by functional localizer. The MC/MI coefficient of 1 is marked by a thicker orange horizontal line to indicate identical response to both kinds of motion. Bars represent the mean coefficients ± standard error of the mean across runs and participants (n indicates number of hemispheres).

To determine the relative preference to motion coherence, we calculated the averaged coefficient of coherency MC/MI (see Methods) in all the studied motion sensitive regions (Fig. 6). A MC/MI coefficient of 1 means that MC and MI have the same effect in a given ROI (marked in the figure by a thicker orange horizontal line).

The coherency coefficients shown in Figure 6A were those obtained by the general contrast M-S for areas CSv, V3A, and IPSmot, and by the localizer for areas V6, MT, and MST+. From data shown in Figure 6A it is evident that V6 and IPSmot were the two areas with the greatest MC/MI coefficient (2.8±0.14 and 2.79±0.14 respectively). This means that coherent stimuli (MC) evoked almost three times the response of incoherent stimuli (MI) in both V6 and IPSmot. In other words, there was a strong preference in these areas for optic flow stimulation that was consistent with egomotion. Since the ROI of V6 by functional localizer is not independent, being obtained with optical flow stimulations, it could be argued that it introduces a bias in the results towards MC stimuli. However, Figure 6B shows that the values obtained with an independent (M-S) functional definition of V6 are nearly the same (t test; t_(12)_ =  −1.547; n.s.), indicating a genuine, not biased strong preference of area V6 for an optic flow stimulation consistent with egomotion.

The MC/MI coefficient in areas V3A and MT is close to 1 (0.92±0.05 and 1.17±0.06, respectively), meaning that these regions respond equally well to both kinds of motion. In area MST+, instead, the MC/MI coefficient is 1.43±0.07, that is the area prefers the motion coherence, but not as much as areas V6 and IPSmot. Figure 6C shows that the coherence value in MT+ (1.27±0.06) is the mathematical average of the two values obtained in MT and MST+. This strengthens the need to separately refers to the two subdivisions to avoid masking effects or lack of significance due to the average which in some cases could cancel out possible differential effects.

The MC/MI coefficient in area CSv is −0.23±0.01, meaning that the MI had no or negative effects in this region.

Interestingly, our results mirror that observed in a recent paper by Cardin and Smith [6] where an egomotion incoherent coefficient (EI/EC, substantially the opposite of our MC/MI coefficient) was used to calculate the sensitivity to egomotion of different brain areas. In line with our results, they found coefficients smaller than 0.5, indicating strong involvement in egomotion, in areas IPSmot and V6, and a coefficient close to 1, indicating no involvement in egomotion, in area V3A. Unlike present results, they found a positive small coefficient in area CSv which would indicate a stronger response in this area for the egomotion. Note however that they did not test a random condition but contrasted two coherent motion conditions. As we found here, CSv region is strongly inhibited by the random motion and this can explain the apparent discrepant results on this area.

## Discussion

Event-related fMRI experiments, 3D motion and wide-field stimulation, functional localizers and brain mapping methods were used to study the sensitivity of six distinct motion areas (V6, MT, MST+, V3A, CSv and IPSmot) to different types of optic flow stimuli. Results show that only areas V6, MST+ and IPSmot are specialized in distinguishing among the various types of flow patterns, whereas areas MT and V3A show no preferences. The visual area CSv, which has recently been shown to be activated by both visual self-motion information [6], [49] and vestibular stimulations [50], surprisingly does not appear to be responsive to the different flow patterns we used. On the other hand, present results show that CSv is strongly inhibited by Random motion and Static visual stimulations, that is by incoherent motion and any type of visual stimulation that have not a vestibular input counterpart in physiological conditions. It could be that in physiological conditions an excitatory vestibular input activates the CSv area during self motion, but we are aware that at present this is only a pure speculation.

The visual motion stimulations used in this experiment were first psychophysically tested on subjects and the behavioral results showed that the four coherent motion conditions are all able to evoke a strong illusory egomotion sensation each along a different plane. Thus hereafter we’ll use indifferently the terms motion or egomotion to refer to our four coherent stimuli (radial, translational, circular and spiral). In the following sections, the results are discussed for each visual area in which discriminative activity between the various types of flow patterns was found.

### Area V6

Our key finding concerns visual area V6, recently identified by our group in humans [26]. Present results show that human V6 codes the motion coherence (Fig. 6A, B), which evokes almost three times the response of random motion in this area. The V6 strong preference for coherent motion confirms previous fMRI studies [5], [6], [51], [52] and also a recent combined VEPs/fMRI works by our group [53] which shows that area V6 is one of the most early stations coding the motion coherence. Present results also reveal that V6 is particularly sensitive to egomotion (compare Fig. 4B with Fig. 2), in particular to the Translational Egomotion.

The selective preference for the 3D translational egomotion observed here is a new result. Among the past fMRI human studies which have investigated the neural correlates of specific optic flow coherent components in humans, only a few tested the region of POs and/or precuneus, finding a generic medial parietal preference for radial [54] or circular [16], [55] components. Conversely, Deutschlander et al. [56] found no differences in the bold signal along the POs between radial and circular components. In agreement with these previous studies, we found a significant response in area V6 for radial and circular motion, when compared to a baseline, and did not find differences between the two types of motion. None of the previous studies however tested the translational motion. A direct comparison between ours and the previous fMRI studies is also limited by the fact that here we are referring specifically to area V6, whereas the previous studies generically refer to a medial parietal portion of cortex that certainly includes several functional regions, among which, likely, also area V6.

### Area IPSmot

Present results show that area IPSmot responds much more to coherent than to incoherent stimuli. Interestingly, the weakest response is observed for random motion, which activates area IPSmot much less than static visual stimulation. Together with V6, IPSmot is the area with the greatest Coherent coefficient (see Fig. 6A). Indeed, as in area V6, the motion coherency evokes almost three times the response of random motion in this area. The egomotion sensitivity we found in area IPSmot confirms previous findings suggesting that this area processes optic flow and egomotion (e.g., [4], [6], [46], [57]. Interestingly, a recent fMRI study found that area VIP is sensitive to the presence of stereoscopic depth gradients associated with self motion [58]. Present data also agree with the functional properties of macaque area VIP, whose neurons respond selectively to optical flow stimuli [59], [60].

### Area MST+

MST+ is able to distinguish between different 3D egomotion, and is more sensitive to Translational and Radial Egomotion than to the Circular one (Fig. 4D). This preference was already observed in the past in both macaque (21,61,62,63,64,65,66,67,68] and human [2], [11], [22], [69]. These past studies led to a general agreement in reporting MST+ as an area sensitive to the motion coherence and to egomotion signals [2], [3], [11], [22], [52], [70]. However, recent studies failed to report positive evidence in favor of a role of MST+ in egomotion (e.g., [3], [4]) raising doubts about its effective importance in terms of egomotion perception. Although present results seem to indicate that MST+ is specialized in distinguishing among different egomotion flow patterns, we expected to find a more selective response profile in this area. In contrast, we found a moderate MST+ preference for the motion coherency (relatively high response to the random condition and coherency coefficient about 1.5; see Fig. 6A and 6C), meaning that this area prefers motion coherence but is not strongly selective for it. Our results indicate that MST+ occupies an *intermediate* position in between areas V6 and IPSmot, that are selective for motion coherence, and MT and V3A, insensitive to the coherence of motion (see Fig. 6A).

The weak selective response profile in area MST+ (and thus its subtle difference with MT) could be caused by the way in which MT and MST+ are defined. Therefore, it is appropriate to issue certain caveats here about the functional localizer introduced by Huk et al [13]. It is possible that MST+ and MT are genuinely different but that the difference is eroded by imperfect separation of the two regions during localization. In fact, according to this localizer only those voxels responding to ipsilateral stimulation are included in the MST+ ROI. Therefore, if the receptive fields of some MST+ neurons include mainly contralateral regions, the voxels containing them may be misclassified as MT, reducing the differences between the two areas.

### Functional Considerations

Present results show that at least three cortical motion areas (V6, IPSmot and MST+) are specialized in distinguishing among different types of self-movement. All these three areas showed an high response for the translational egomotion, which was maximum in V6 and IPSmot and less marked in MST+. The behavioral results showed that the four coherent motion conditions tested here evoked a comparable egomotion sensation. Thus, the high response to the translational motion observed in areas V6, IPSmot and MST+ cannot be explained by a bias in the visual stimuli construction, neither by a specific sensitivity of these areas to vection (which is constant). Therefore, the high response of these three motion areas to the translational motion raises the question of its functional significance.

The translational motion condition used here simulate an observer translating horizontally, such as for example when we are on a moving train while looking on the lateral window. In physiologic conditions, during body translation in the horizontal plane the retinal motion of objects located at different distances respect to the observer generates the *differential motion parallax* which is the perceived difference in *speed* and *direction* of nearby objects compared to far away ones. As we move, objects that are closer to us move faster across our field of view than do objects that are in the distance. Moreover, objects that are closer or distant respect to the fixation point move in opposite or same direction, respectively, with respect to our motion. This latter effect is present only in the translational motion. Therefore, the differential motion parallax is a powerful depth cue that results mainly from our *translational* motion and that enables us to evaluate the relative distance of near and far objects in the environment.

Traslational flow is thus conceptually different with respect to the other flow patterns. The spiral and radial 3D flow stimuli used here, for instance, produced the effect of navigating through a field of stars, heading towards a particular point on the screen (the focus of expansion). In the translational optic flow, in contrast, the accent is not on the heading direction but on the lateral visual flow produced by near and far external objects. Note also that only in the translational flow each individual dot maintains a constant size and speed with respect to the observer conveying information about their distance, while in the radial or spiral flow each dot moves forward or inward with a progressive increasing or reduction of the size and speed of each dot. This latter feature is more related to the focus of expansion of the flow pattern and thus is more informative about heading.

Therefore the translational stimulus gives the possibility to evaluate the depth of objects in a dynamic condition such as that created by self motion. The strong response to translational motion observed particularly in areas V6 and IPSmot suggests that these areas process visual egomotion signals to analyze the 3D layout of the environment and to extract information about the relative distance of objects located in it, likely in order to act on them, or to avoid them. In fact, V6 is strongly connected with the neighbouring visuomotor area V6A [71], [72], [73], and V6A in turn is strongly involved in encoding depth for eye and arm movements [74,75,76). We can suppose that information on objects in depth which are translating in space because of the self motion are processed in V6 and conveyed to V6A for evaluating object distance so to orchestrate the eye and arm movements. Even though areas V6 and VIP are not directly involved in the control of movement, their outputs are known to converge on the dorsal and ventral premotor cortices, respectively [77], [78], [79], [80], [81]. In turn, the premotor cortex controls the direction of arm movements toward objects in the peripersonal space. Since V6A mainly represents the upper limbs [82] and VIP the face [83] they could be involved in processing visual information for acting in the whole peripersonal space.

Macaque data suggest that V6 is involved in object-motion recognition. The hypothesis is based on the fact that V6 contains a high percentage of *real-motion cells*, that is, cells activated by the actual movement of an object in the visual field, regardless of the movement of object retinal images induced by the eye movements [84]. Likely, this type of cells allows detection and recognition of real movement in the visual field, even in such critical situations as when the retinal images are continuously in motion because of self-motion [84]. This process is essential both for the avoidance of obstacles and for planning the handling of nearby objects. In summary, present data and the above considerations suggest that V6 is involved in both object and self-motion recognition. It could be involved in distinguishing object-motion from self-motion and in providing information about location in space of moving and static objects during self-motion, particularly in a dynamically unstable environment.

A final note goes to area MST+ and its possible functional role with respect to areas V6 and IPSmot. Here we show that MST+ is able to distinguish between different 3D flow fields, which is a necessary prerequisite for an area processing egomotion signals (e.g., [65]). Unlike V6 and IPSmot, MST+ shows a similar sensitivity for translational and radial flow, strongly suggesting, in line with previous studies (e.g., [14]), the involvement of this area in the processing of heading direction. The MST+ contribution to the locomotion finds also support in macaque studies, reporting that MSTd neurons signal the direction of heading during self-motion (e.g., [84]). MST could be an intermediate neural stage in the egomotion perception that convey direction of heading signals to higher and more specialized neural stations of the dorsal pathway.

## References

[1] Tootell RB, Reppas JB, Kwong KK, Malach R, Born RT, et al (1995) Functional analysis of human MT and related visual cortical areas using magnetic resonance imaging. J Neurosci 15: 3215–3230. 7722658 10.1523/JNEUROSCI.15-04-03215.1995 PMC6577785

[2] Morrone MC, Tosetti M, Montanaro D, Fiorentini A, Cioni G, et al (2000) A cortical area that responds specifically to optic flow, revealed by fMRI. Nature Neurosci 3: 1322–1328. 11100154 10.1038/81860

[3] Kleinschmidt A, Thilo KV, Büchel C, Gresty MA, Bronstein AM, et al (2002) Neural correlates of visual-motion perception as object- or self-motion. Neuroimage 16(4): 873–882. 12202076 10.1006/nimg.2002.1181

[4] Wall MB, Smith AT (2008) The Representation of Egomotion in the Human Brain. Curr Biol 18: 191–194. 18221876 10.1016/j.cub.2007.12.053

[5] Pitzalis S, Sereno MI, Committeri G, Fattori P, Galati G, et al (2010) Human V6: the medial motion area. Cerebral Cortex 20(2): 411–24. 19502476 10.1093/cercor/bhp112 PMC2803738

[6] Cardin V, Smith AT (2010) Sensitivity of human visual and vestibular cortical regions to egomotion-compatible visual stimulation. Cereb Cortex 20 (8): 1964–73. 10.1093/cercor/bhp268 PMC2901022 20034998

[7] Gibson JJ (1966) The senses considered as perceptual systems. Houghton Mifflin, Boston.

[8] Koenderink JJ (1986) Optic flow. Vision Res 26: 161–168. 3716209 10.1016/0042-6989(86)90078-7

[9] Hixson WC, Niven JI, Correia MJ (1966) Kinematics nomenclature for psychological acceleration. Monograph 14. Pensacola, FL: Navel Aerospace Medical Institute.

[10] Beer J, Blakemore C, Previc FH, Liotti M (2002) Areas of the human brain activated by ambient visual motion, indicating three kinds of self-movement. Exp Brain Res 143(1): 78–88. 11907693 10.1007/s00221-001-0947-y

[11] Smith T, Wall MB, Williams AL, Singh KD (2006) Sensitivity to optic flow in human cortical areas MT and MST. Eur J Neurosci 23: 561–569. 16420463 10.1111/j.1460-9568.2005.04526.x

[12] Dukelow SP, DeSouza JF, Culham JC, van den Berg AV, Menon RS, et al (2001) Distinguishing subregions of the human MT+ complex using visual fields and pursuit eye movements. J Neurophysiol 86(4): 1991–2000. 11600656 10.1152/jn.2001.86.4.1991

[13] Huk AC, Dougherty RF, Heeger DJ (2002) Retinotopy and functional subdivision of human areas MT and MST. J Neurosci 22: 7195–7205. 12177214 10.1523/JNEUROSCI.22-16-07195.2002 PMC6757870

[14] Cardin V, Hemsworth L, Smith AT (2012) Adaptation to heading direction dissociates the roles of human MST and V6 in the processing of optic flow. J Neurophysiol May 16. 10.1152/jn.00002.2012 PMC3424094 22592304

[15] Cheng K, Fujita H, Kanno I, Miura S, Tanaka K (1995) Human cortical regions activated by wide-field visual motion: an H2 150 PET study. J Neurophysiol 74: 413–427. 7472342 10.1152/jn.1995.74.1.413

[16] Previc FH, Liott M, Blakemore C, Beer J, Fox P (2000) Functional imaging of brain areas involved in the processing of coherent and incoherent wide field-of-view visual motion. Exp Brain Res 131(4): 393–405. 10803409 10.1007/s002219900298

[17] Galletti C, Fattori P, Gamberini M, Kutz DF (1999) The cortical visual area V6: brain location and visual topography. Eur J Neurosci 11: 3922–3936. 10583481 10.1046/j.1460-9568.1999.00817.x

[18] Priebe NJ, Lisberger SG (2004) Estimating target speed from the population response in visual area MT. J Neurosci 24: 1907–1916. 14985431 10.1523/JNEUROSCI.4233-03.2004 PMC2553806

[19] Nover H, Anderson CH, DeAngelis GC (2005) A logarithmic, scale invariant representation of speed in macaque middle temporal area accounts for speed discrimination performance. J Neurosci 25: 10049–10060. 16251454 10.1523/JNEUROSCI.1661-05.2005 PMC6725555

[20] Liu J, Newsome WT (2006) Local field potential in cortical area MT: stimulus tuning and behavioral correlations. J Neurosci 26: 7779–7790. 16870724 10.1523/JNEUROSCI.5052-05.2006 PMC6674213

[21] Nelissen K, Vanduffel W, Orban GA (2006) Charting the lower superior temporal region, a new motion-sensitive region in monkey superior temporal sulcus. J Neurosci. May 31 26(22): 5929–47. 10.1523/JNEUROSCI.0824-06.2006 PMC6675228 16738235

[22] Wall MB, Lingnau A, Ashida H, Smith AT (2008) Selective visual responses to expansion and rotation in the human MT complex revealed by functional magnetic resonance imaging adaptation. Eur J Neurosci 27: 2747–2757. 18547254 10.1111/j.1460-9568.2008.06249.x

[23] Kolster H, Peeters R, Orban GA (2010) The retinotopic organization of the human middle temporal area MT/V5 and its cortical neighbors. J Neurosci 30(29): 9801–20. 20660263 10.1523/JNEUROSCI.2069-10.2010 PMC6632824

[24] Sereno MI, Dale AM, Reppas JB, Kwong KK, Belliveau JW, et al (1995) Borders of multiple visual areas in humans revealed by functional magnetic resonance imaging. Science 268: 889–893. 7754376 10.1126/science.7754376

[25] Tootell RB, Mendola JD, Hadjikhani NK, Ledden PJ, Liu AK, et al (1997) Functional analysis of V3A and related areas in human visual cortex. J Neurosci 17: 7076–7078. 10.1523/JNEUROSCI.17-18-07060.1997 PMC6573277 9278542

[26] Pitzalis S, Galletti C, Huang RS, Patria F, Committeri G, et al (2006) Wide-field retinotopy defines human cortical visual area V6. J Neurosci 26: 7962–7973. 16870741 10.1523/JNEUROSCI.0178-06.2006 PMC6674231

[27] Kwong KK, Belliveau JW, Chesler DA, Goldberg IE, Weisskoff RM, et al (1992) Dynamic magnetic resonance imaging of human brain activity during primary sensory stimulation. Proc Natl Acad Sci USA 89(12): 5675–9. 1608978 10.1073/pnas.89.12.5675 PMC49355

[28] de Jong BM, Shipp S, Skidmore B, Frackowiak RS, Zeki S (1994) The cerebral activity related to the visual perception of forward motion in depth. Brain 117: 1039–1054. 7953587 10.1093/brain/117.5.1039

[29] Kennedy RS, Hettinger LJ, Harm DL, Ordy JM, Dunlap WP (1996) Psychophysical scaling of circular vection (CV) produced by optokinetic (OKN) motion: individual differences and effects of practice. J Vestib Res 6(5): 331–41. 8887891

[30] Dale AM, Fischl B, Sereno MI (1999) Cortical surface-based analysis. I. Segmentation and surface reconstruction. Neuroimage 9: 179–194. 9931268 10.1006/nimg.1998.0395

[31] Fischl B, Sereno MI, Dale AM (1999) Cortical surface-based analysis: II: inflation, flattening, and a surface-based coordinate system. Neuroimage, 9, 195–207. 10.1006/nimg.1998.0396 9931269

[32] Friston KJ, Ashburner J, Poline JB, Frith CD, Heather JD, et al (1995) Spatial registration and normalization of images. Hum Brain Mapp 2: 165–189.

[33] Mazziotta JC, Toga AW, Evans A, Fox P, Lancaster J (1995) A probabilistic atlas of the human brain: theory and rationale for its development. The International Consortium for Brain Mapping (ICBM). NeuroImage 2: 89–101. 9343592 10.1006/nimg.1995.1012

[34] Talairach J, Tournoux P (1988) Co-planar stereotaxic atlas of the human brain. New York: Thieme.

[35] Van Essen DC (2005) A Population-Average, Landmark- and Surface-based (PALS) atlas of human cerebral cortex. Neuroimage 28(3): 635–62. 16172003 10.1016/j.neuroimage.2005.06.058

[36] Lancaster JL, Woldorff MG, Parsons LM, Liotti M, Freitas CS, et al (2000) Automated Talairach atlas labels for functional brain mapping. Hum Brain Mapp 10(3): 120–31. 10912591 10.1002/1097-0193(200007)10:3<120::AID-HBM30>3.0.CO;2-8 PMC6871915

[37] Tzourio-Mazoyer N, Landeau B, Papathanassiou D, Crivello F, Etard O, et al (2002) Automated anatomical labeling of activations in SPM using a macroscopic anatomical parcellation of the MNI MRI single-subject brain. Neuroimage 15(1): 273–89. 11771995 10.1006/nimg.2001.0978

[38] Castriota-Scanderbeg A, Hagberg GE, Cerasa A, Committeri G, Galati G, et al (2005) The appreciation of wine by sommeliers: a functional magnetic resonance study of sensory integration. Neuroimage 25: 570–578. 15784436 10.1016/j.neuroimage.2004.11.045

[39] Galati G, Committeri G, Spitoni G, Aprile T, Di Russo F, et al (2008) A selective representation of the meaning of actions in the auditory mirror system. Neuroimage 40: 1274–1286. 18276163 10.1016/j.neuroimage.2007.12.044

[40] Galati G, Committeri G, Pitzalis S, Pelle G, Patria F, et al (2011) Intentional signals during saccadic and reaching delays in the human posterior parietal cortex. Eur J Neurosci 34(11): 1871–85. 22017280 10.1111/j.1460-9568.2011.07885.x

[41] Ionta S, Heydrich L, Lenggenhager B, Mouthon M, Fornari E, et al (2011) Multisensory mechanisms in temporoparietal cortex support self-location and first-person perspective. Neuron 70: 363–374. 21521620 10.1016/j.neuron.2011.03.009

[42] Kriegeskorte N, Simmons WK, Bellgowan PS, Baker CI (2009) Circular analysis in systems neuroscience: the dangers of double dipping. Nat Neurosci 12(5): 535–40. 19396166 10.1038/nn.2303 PMC2841687

[43] Hagler DJ Jr, Sereno MI (2006) Spatial maps in frontal and prefrontal cortex. Neuroimage 29: 567–577. 16289928 10.1016/j.neuroimage.2005.08.058

[44] Bremmer F, Schlack A, Shah NJ, Zafiris O, Kubischik M, et al.. (2001) Polymodal Motion Processing in Posterior Parietal and Premotor Cortex: a human fMRI study strongly implies equivalencies between humans and monkeys. Neuron 29(1), 287–296. 10.1016/s0896-6273(01)00198-2 11182099

[45] Bartels A, Zeki S, Logothetis NK (2008) Natural vision reveals regional specialization to local motion and to contrast-invariant, global flow in the human brain. Cereb Cortex. Mar;18(3): 705–17. Epub 2007 Jul 5. 10.1093/cercor/bhm107 17615246

[46] Sereno MI, Huang RS (2006) A human parietal face area contains aligned head-centered visual and tactile maps. Nat Neurosci 9(10): 1337–43. 16998482 10.1038/nn1777

[47] Watson JD, Myers R, Frackowiak RS, Hajnal JV, Woods RP, et al (1993) Area V5 of the human brain: evidence from a combined study using positron emission tomography and magnetic resonance imaging. Cereb Cortex 3: 79–94. 8490322 10.1093/cercor/3.2.79

[48] Tootell RB, Hadjikhani N, Hall EK, Marrett S, Vanduffel W, et al (1998) The retinotopy of visual spatial attention. Neuron 21: 1409–1422. 9883733 10.1016/s0896-6273(00)80659-5

[49] Fischer E, Bülthoff HH, Logothetis NK, Bartels A (2012) Visual motion responses in the posterior cingulate sulcus: a comparison to V5/MT and MST. Cereb Cortex 22(4): 865–76. 21709176 10.1093/cercor/bhr154 PMC3306574

[50] Smith AT, Wall MB, Thilo KV (2012) Vestibular inputs to human motion-sensitive visual cortex. Cereb Cortex 22(5): 1068–77. 21743097 10.1093/cercor/bhr179

[51] von Pföstl V, Stenbacka L, Vanni S, Parkkonen L, Galletti C, et al (2009) Motion sensitivity of human V6: a magnetoencephalography study. Neuroimage 45(4): 1253–63. 19211036 10.1016/j.neuroimage.2008.12.058

[52] Helfrich RF, Becker HG, Haarmeier T (2012) Processing of Coherent Visual Motion in Topographically Organized Visual Areas in Human Cerebral Cortex. Brain Topogr Apr 19. 10.1007/s10548-012-0226-1 22526896

[53] Pitzalis S, Bozzacchi C, Bultrini A, Fattori P, Galletti C, et al (2013) Parallel motion signals to the medial and lateral motion areas V6 and MT+. Neuroimage 67: 89–100. 23186916 10.1016/j.neuroimage.2012.11.022

[54] Ptito M, Kupers R, Faubert J, Gjedde A (2001) Cortical representation of inward and outward radial motion in man. Neuroimage 14(6): 1409–1415. 11707096 10.1006/nimg.2001.0947

[55] Brandt T, Bucher SF, Seelos KC, Dieterich M (1998) Bilateral functional MRI activation of the basal ganglia and middle temporal/medial superior temporal motion-sensitive areas: optokinetic stimulation in homonymous hemianopia. Arch Neurol 55(8): 1126–1131. 9708964 10.1001/archneur.55.8.1126

[56] Deutschländer A, Bense S, Stephan T, Schwaiger M, Dieterich M, et al (2004) Rollvection versus linearvection: comparison of brain activations in PET. Hum Brain Mapp 21(3): 143–53. 14755834 10.1002/hbm.10155 PMC6871853

[57] Peuskens H, Sunaert S, Dupont P, Van Hecke P, Orban GA (2001) Human brain regions involved in heading estimation. J Neurosci 21(7): 2451–61. 11264319 10.1523/JNEUROSCI.21-07-02451.2001 PMC6762416

[58] Cardin V, Smith AT (2011) Sensitivity of human visual cortical area V6 to stereoscopic depth gradients associated with self-motion. J Neurophysiol 106(3): 1240–9. 21653717 10.1152/jn.01120.2010 PMC3174812

[59] Colby CL, Duhamel JR, Goldberg ME (1993) Ventral intraparietal area of the macaque: anatomic location and visual response properties. J Neurophysiol 69: 902–914. 8385201 10.1152/jn.1993.69.3.902

[60] Bremmer F, Duhamel JR, Ben Hamed S, Graf W (2002) Heading encoding in the macaque ventral intraparietal area (VIP). Eur J Neurosci 16: 1554–1568. 12405970 10.1046/j.1460-9568.2002.02207.x

[61] Saito H, Yukie M, Tanaka K, Hikosaka K, Fukada Y, et al (1986) Integration of direction signals of image motion in the superior temporal sulcus of the macaque monkey. J Neurosci 6(1): 145–57. 3944616 10.1523/JNEUROSCI.06-01-00145.1986 PMC6568620

[62] Tanaka K, Saito H (1989) Analysis of motion of the visual field by direction, expansion/contraction, and rotation cells clustered in the dorsal part of the medial superior temporal area of the macaque monkey. J Neurophysiol 62(3): 626–41. 2769351 10.1152/jn.1989.62.3.626

[63] Tanaka K, Fukada Y, Saito H (1989) Underlying mechanisms of the response specificity of expansion/contraction and rotation cells in the dorsal part of the MST area of the macaque monkey. J Neurophysiol 62: 642–656. 2769352 10.1152/jn.1989.62.3.642

[64] Duffy CJ, Wurtz RH (1991) Sensitivity of MST neurons to optic flow stimuli. I. A continuum of response selectivity to large-field stimuli. J Neurophysiol 65(6): 1329–45. 1875243 10.1152/jn.1991.65.6.1329

[65] Duffy CJ (1998) MST neurons respond to optic flow and translational movement. J Neurophysiol 80(4): 1816–27. 9772241 10.1152/jn.1998.80.4.1816

[66] Orban GA, Lagae L, Verri A, Raiguel S, Xiao D, et al (1992) First-order analysis of optical flow in monkey brain. Proc Natl Acad Sci U S A 89(7): 2595–9. 1557363 10.1073/pnas.89.7.2595 PMC48708

[67] Graziano MS, Andersen RA, Snowden RJ (1994) Tuning of MST neurons to spiral motions. J Neurosci 14(1): 54–67. 8283251 10.1523/JNEUROSCI.14-01-00054.1994 PMC6576843

[68] Eifuku S, Wurtz RH (1998) Response to motion in extrastriate area MSTl: center-surround interactions. J Neurophysiol 80(1): 282–96. 9658050 10.1152/jn.1998.80.1.282

[69] Kovács G, Raabe M, Greenlee MW (2008) Neural correlates of visually induced self-motion illusion in depth. Cereb Cortex 18: 1779–1787. 18063566 10.1093/cercor/bhm203

[70] Galletti C, Gamberini M, Kutz DF, Fattori P, Luppino G, et al (2001) The cortical connections of area V6: an occipito-parietal network processing visual information. Eur J Neurosci 13: 1572–1588. 11328351 10.1046/j.0953-816x.2001.01538.x

[71] Gamberini M, Passarelli L, Fattori P, Zucchelli M, Bakola S, et al (2009) Cortical connections of the visuomotor parietooccipital area V6Ad of the macaque monkey. J Comp Neurol 513(6): 622–642. 19235224 10.1002/cne.21980

[72] Passarelli L, Rosa MG, Gamberini M, Bakola S, Burman KJ, et al (2011) Cortical connections of area V6Av in the macaque: a visual-input node to the eye/hand coordination system. J Neurosci 31(5): 1790–801. 21289189 10.1523/JNEUROSCI.4784-10.2011 PMC6623732

[73] Hadjidimitrakis K, Breveglieri R, Placenti G, Bosco A, Sabatini SP, et al (2011) Fix your eyes in the space you could reach: neurons in the macaque medial parietal cortex prefer gaze positions in peripersonal space. PLoS One 6(8): e23335. 21858075 10.1371/journal.pone.0023335 PMC3157346

[74] Hadjidimitrakis K, Breveglieri R, Bosco A, Fattori P (2012) Three-dimensional eye position signals shape both peripersonal space and arm movement activity in the medial posterior parietal cortex. Front Integr Neurosci 6: 37. 22754511 10.3389/fnint.2012.00037 PMC3385520

[75] Breveglieri R, Hadjidimitrakis K, Bosco A, Sabatini SP, Galletti C, et al (2012) Eye position encoding in three-dimensional space: integration of version and vergence signals in the medial posterior parietal cortex. J Neurosci 32(1): 159–69. 22219279 10.1523/JNEUROSCI.4028-11.2012 PMC6621321

[76] Shipp S, Blanton M, Zeki S (1998) A visuo-somatomotor pathway through superior parietal cortex in the macaque monkey: cortical connections of areas V6 and V6A. Eur J Neurosci 10(10): 3171–3193. 9786211 10.1046/j.1460-9568.1998.00327.x

[77] Luppino G, Murata A, Govoni P, Matelli M (1999) Largely segregated parietofrontal connections linking rostral intraparietal cortex (areas AIP and VIP) and the ventral premotor cortex (areas F5 and F4). Exp Brain Res 128: 181–187. 10473756 10.1007/s002210050833

[78] Lewis JW, Van Essen D (2000) Corticocortical connections of visual, sensorimotor, and multimodal processing areas in the parietal lobe of the macaque monkey. J Comp Neurol 428: 112–137. 11058227 10.1002/1096-9861(20001204)428:1<112::aid-cne8>3.0.co;2-9

[79] Tanne-Gariepy J, Rouiller EM, Boussaoud D (2002) Parietal inputs to dorsal versus ventral premotor areas in the macaque monkey: evidence for largely segregated visuomotor pathways. Exp Brain Res 145: 91–103. 12070749 10.1007/s00221-002-1078-9

[80] Galletti C, Fattori P, Gamberini M, Kutz DF (2004) The most direct visual pathway to the frontal cortex. Cortex 40(1): 216–7. 15070012 10.1016/s0010-9452(08)70956-0

[81] Breveglieri R, Kutz DF, Fattori P, Gamberini M, Galletti C (2002) Somatosensory cells in the parieto-occipital area V6A of the macaque. Neuroreport 13 (16): 2113–2116. 10.1097/00001756-200211150-00024 12438936

[82] Duhamel JR, Colby CL, Goldberg ME (1998) Ventral intraparietal area of the macaque: congruent visual and somatic response properties. J Neurophysiol 79(1): 126–36. 9425183 10.1152/jn.1998.79.1.126

[83] Galletti C, Fattori P (2003) Neuronal mechanisms for detection of motion in the field of view. Neuropsychologia 41: 1717–1727. 14527536 10.1016/s0028-3932(03)00174-x

[84] Grossberg S, Mingolla E, Pack C (1999) A neural model of motion processing and visual navigation by cortical area MST. Cereb Cortex 9(8): 878–95. 10601006 10.1093/cercor/9.8.878

